# Innovative Strategies and Recent Advances in Liver Surgery

**DOI:** 10.1155/2013/517279

**Published:** 2013-04-02

**Authors:** Andrea Lauterio, Irinel Popescu, Juan Carlos García-Valdecasas, Luciano De Carlis

**Affiliations:** ^1^Dipartimento di Chirurgia Generale e Trapianti Addominali, Ospedale Niguarda, Piazza Ospedale Maggiore, 3 20162 Milano, Italy; ^2^Center of General Surgery and Liver Transplantation, Fundeni Clinical Institute, Bucharest, Romania; ^3^Department of Surgery, Hospital Clínic, University of Barcelona, Barcelona, Catalunya, Spain

Techniques for hepatic surgery have evolved over the past few decades and have broadened indications for liver resection (LR) for liver tumors. New strategies including downsizing chemotherapy, two-stage LR with or without portal vein embolization, and resection combined with ablative methods allow tailoring the treatment to each patient depending on condition of the liver and tumor burden. In the recent years, the new dissector devices have been developed and together with the use of intraoperative ultrasound allow a new approach to the anatomical ultrasound-guided liver resection, even for large tumors located in challenging positions.

Improvements in imaging evaluation with high-resolution CT scan or MRI allow new methods for the study of the future remnant liver and play an important role in the planning of the resection strategy reducing the risk of major complications and liver failure, especially in patients who undergo major resection. In addition, development of new technology in local ablative therapies for liver tumors is posing a competition to LR.

The incidence of hepatocellular carcinoma (HCC) is climbing rapidly and in a current climate of organ shortage has led to the re-evaluation of locoregional therapies and resectional surgery to manage the case load. The introduction of biological therapies has had a new dimension to care, adding to the complexities of multidisciplinary team working in the management of HCC. S. E. Khorsandi and N. Heaton give a very comprehensive overview of the present day management strategies and decision making for patients with HCC.

Simultaneous resection of primary colorectal carcinoma (CRC) and synchronous liver metastases (SLM) is subject of debate with respect to morbidity in comparison to staged resection. In contrast to the extensive literature on staged laparoscopic colorectal and laparoscopic liver surgery, there are only a few reports on combined laparoscopic colorectal and liver resection.

L. T. Hoekstra and colleagues report their initial experience of simultaneous laparoscopic resection of primary CRC and SLM. According to the modern literature, the authors conclude that patient selection and expertise are essential for this complex type of surgery and the multidisciplinary team should decide on optimal timing within multimodality schedules.

I. Popescu and S. T. Alexandrescu challenge recent evidence in the different surgical options for initially unresectable colorectal liver metastases. The authors illustrate the available oncosurgical modalities including liver resection following portal vein ligation/embolization, “two-stage” liver resection, one-stage ultrasonically guided liver resection, hepatectomy following conversion chemotherapy, and liver resection combined with thermal ablation. The authors discuss the role of liver transplantation (LT) as a future opportunity in the treatment of unresectable CRLM in selected patients, taking into account the related ethical considerations especially in case of LT from living donor or LT with marginal grafts. Although the available data do not support liver transplantation as a routine procedure in patients with CRLM, this paper could promote the debate on this issue.

Partial liver transplantation, including split-liver and living donor liver transplantation, represents another important application of these advantages applied in the field of organ transplantation. 

N. Akamatsu and Y. Sugawara provide a review article on the current trends and controversies in living donor liver transplantation (LDLT) for patients with HCV in relation to the perspectives from deceased donor.

They focused their attention on the recent advances in antiviral treatment for the recurrent hepatitis C after LT reporting the different strategies from the Japanese LDLT centers.

Minimally invasive approach in the field of HPB surgery is gaining popularity due to the availability of new laparoscopic instruments for liver transection. Laparoscopic LR has evolved significantly over the past decade moving from an experimental procedure to a standard part of the hepatic surgeon's armamentarium. Most recently, robotic-assisted technology offers solutions to overcome the limitations of conventional laparoscopic resection.

With the review paper titled *“Laparoscopy in liver transplantation: The future has arrived*,*”* Q. Lai and colleagues shed further light on the role of the laparoscopy in this field of surgery. Intent of the review is to underline the current role of diagnostic and therapeutic laparoscopy in patients waiting for LT, in the living donor LT and in LT recipients.

G. Dapri and colleagues report their initial experience with single-incision transumbilical laparoscopic liver resection (SITLLR) with a detailed technical paper and discuss the future of this approach in terms of indications, potential benefits, and limitations in comparison with multiport laparoscopic technique.

Single incision transumbilical laparoscopy represents the latest advance of the laparoscopic approach; however, its use in LR still remains limited to small reported series, and further evaluation is required to assess the potential advantages and the improvement in the patients outcome. As reported by the authors, at this point of the experience, several questions on SITLLR remain to be addressed, concerning the feasibility and mostly the reproducibility of this technique, the indications, selection criteria, limitations, effect on postoperative outcomes, and long-term results.

F. Romano et al. provide a summarizing paper illustrating methods to prevent bleeding in hepatic surgery. This is of particular interest, as bleeding in HPB surgery represents one of the most common features associated with poor outcome. The paper is based on the literature information and author's experience, and the aim of the study is to investigate the principal solutions to the problem of high blood loss in LR focusing on technological approach to the parenchyma transection.

The aim of this special issue is to update and promote interchange of the current knowledge and recent progress focusing on innovative strategies and recent advances in liver surgery. These manuscripts represent an exciting and insightful snapshot of the recent advances in liver surgery. State-of-the-art, existing challenges, and emerging future topics are highlighted in this special issue, which may inspire the reader and help advance in this field of surgery. We would like to thank all the authors and reviewers for making this special issue in HPB surgery possible.

*Andrea Lauterio* *Andrea Lauterio* *Irinel Popescu* *Irinel Popescu* *Juan Carlos García-Valdecasas* *Juan Carlos García-Valdecasas* *Luciano De Carlis* *Luciano De Carlis*

